# Bacterial motility in rhizosphere colonization: mechanisms, constraints, and implications for microbial inoculants

**DOI:** 10.1093/ismejo/wrag236

**Published:** 2026-09-10

**Authors:** Tong Wang, Brajesh K Singh, Pankaj Trivedi, Zahra F Islam, Ji-Zheng He, Hang-Wei Hu

**Affiliations:** School of Agriculture, Food and Ecosystem Sciences, Faculty of Science, The University of Melbourne, Parkville, Victoria 3010, Australia; UWA School of Agriculture and Environment, The University of Western Australia, Perth, Western Australia 6009, Australia; Institute of Genomics for Crop Abiotic Stress Tolerance (IGCAST), Department of Plant and Soil Science, Texas Tech University, Lubbock, TX 79409, United States; School of Agriculture, Food and Ecosystem Sciences, Faculty of Science, The University of Melbourne, Parkville, Victoria 3010, Australia; School of Agriculture, Food and Ecosystem Sciences, Faculty of Science, The University of Melbourne, Parkville, Victoria 3010, Australia; School of Agriculture, Food and Ecosystem Sciences, Faculty of Science, The University of Melbourne, Parkville, Victoria 3010, Australia

**Keywords:** biofertilizer, sustainable agriculture, chemotaxis, movement ecology, microbiome engineering

## Abstract

Although the potential of microbial inoculants for sustainable agriculture and environmental restoration has been widely recognized, their field performance remains highly variable and often unpredictable. Current research and development frameworks for microbial inoculants primarily focus on their plant growth–promoting functions and metabolic traits, often overlooking the ecological processes that determine whether introduced strains can successfully disperse, access, and establish within the rhizosphere. Increasing evidence suggests that successful dispersal and establishment cannot be assumed in the highly heterogeneous conditions of soil systems. Here, we summarize the key mechanisms underlying bacterial motility and discuss its role within the broader framework of microbial dispersal, highlighting how motility-mediated processes contribute to rhizosphere colonization. We propose that bacterial motility represents a key mechanistic determinant of biofertilizer efficacy. Its role extends beyond the ability of inoculant strains to physically reach the rhizosphere, encompassing competitive colonization on the root surface, long-term persistence, and the ability to respond to dynamic root-derived chemical gradients associated with newly developing root tissues. We argue that inoculant motility should be elevated from a passive descriptive trait to a core design parameter that can be systematically incorporated and regulated during the development and optimization of microbial inoculants. We outline a multi-tiered strategic framework for next-generation biofertilizer engineering that integrates strain selection, community design, motility regulation, and deployment strategies, thereby unlocking the full potential of synthetic microbial consortia for sustainable agriculture, ecosystem restoration, and climate change mitigation.

## Introduction: the colonization bottleneck in biofertilizers

The global population is projected to exceed 10 billion by the end of the twenty-first century [1], placing unprecedented pressure on global food systems. To meet the rising demand for food, conventional agriculture has long relied on the intensive application of chemical fertilizers to boost crop yields and support agricultural and economic development [2]. Although this strategy has delivered substantial yield gains, excessive use of chemical fertilizers has also resulted in a range of environmental problems, largely driven by low nutrient-use efficiency, soil degradation, and biodiversity loss [3]. Moreover, the increasing frequency and intensity of extreme climatic events such as heatwaves, drought, and flooding, along with recurrent pest and disease outbreaks, have further destabilized agricultural production systems [4, 5]. Under these pressures, technological innovations that support sustainable and resilient agricultural practices have become essential to addressing the emerging global food challenge.

Biofertilizers encompass a broad range of products, including organic amendments, microbial metabolites, and living microorganisms. In this perspective, we focus specifically on microbial inoculants, which consist of beneficial microorganisms applied to seeds, plants, or soil to enhance soil biological fertility and crop productivity through diverse biochemical processes [6]. The global biofertilizer market is projected to grow from USD 3.72 billion in 2025 to nearly USD 6 billion by 2030 (an increase of ~60%), at an annual growth rate of over 10%, with North America and Europe holding the largest shares and the Asia-Pacific region showing the fastest growth [7]. As part of biofertilizers, plant growth–promoting rhizobacteria (PGPR) are commonly employed to boost plant growth, improve tolerance to abiotic stressors, and induce systemic resistance against pathogens [8, 9]. Despite their promise, the field performance of biofertilizers remains highly inconsistent, which is largely attributable to the complexity and heterogeneity of soil environments, the low colonization efficiency of introduced microbial strains, and competitive exclusion by resident microbial communities occupying similar ecological niches [10, 11].

Root colonization by microbial inoculants is a multi-stage ecological process that includes movement toward the rhizosphere, successful establishment on root surfaces through interactions and competition with resident or seed-derived microbiota, population persistence and drift, and the expression of plant-beneficial functional traits [9]. The first stage involves two complementary components of microbial dispersal: passive dispersal, the transport of cells by external vectors such as water, fungal hyphae and soil fauna, and active dispersal, self-generated movement driven by the cell’s own motility machinery. Throughout this article, “motility” refers to the active component. These dispersal processes determine whether introduced bacteria can reach the vicinity of the root, after which motility enables short-range navigation through the heterogeneous soil matrix, responses to dynamic chemical gradients generated by root exudates, and the final approach to root surfaces that may already be colonized by resident microbiota [9, 12]. However, current biofertilizer design and evaluation frameworks primarily emphasize static biochemical and structural traits, such as nitrogen fixation, indole-3-acetic acid (IAA) production, phosphate solubilization capacity, stress tolerance, and biofilm formation [13], but largely overlook microbial motility, a functional attribute that may play a pivotal role in field performance (Fig. 1A). The rhizosphere is characterized by complex physical constraints, including discontinuous water films, tortuous pore networks, and solid–liquid interfaces surrounding root hairs and exudate hotspots, all of which impose strong limitations on microbial migration and colonization [14, 15]. In such a constrained semi-solid environment, microorganisms must integrate multiple modes of motility to achieve effective root surface approach and colonization (Fig. 1B). Therefore, we argue that microbial motility represents a critical yet underappreciated link between plant signalling and rhizosphere colonization efficiency.

**Figure 1:**
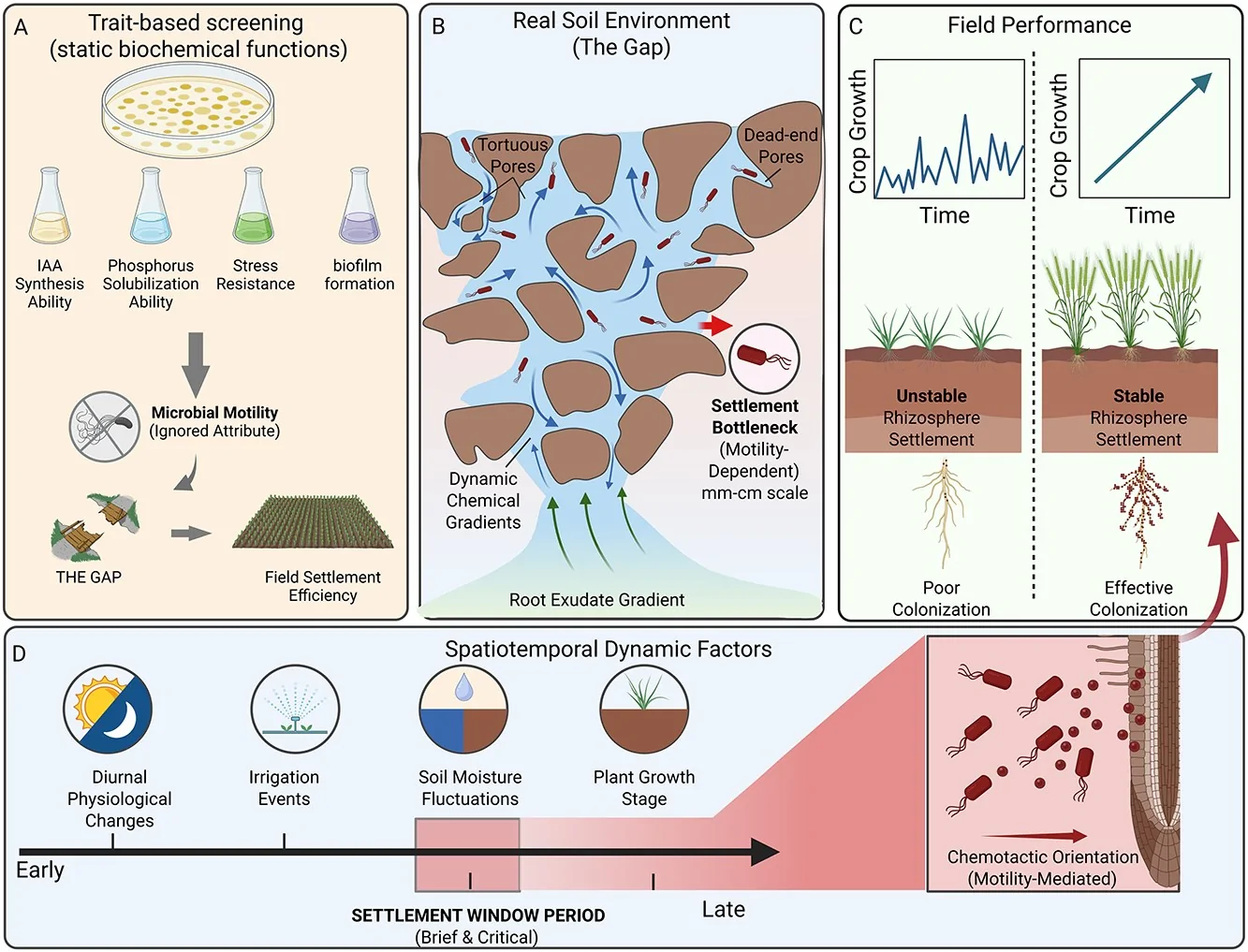
The motility-dependent colonization bottleneck underlying inconsistent biofertilizer performance. (A) Current biofertilizer development pipelines prioritize static biochemical and structural traits, including indole-3-acetic acid (IAA) production, phosphate solubilization, stress resistance, and biofilm formation, but largely overlook microbial motility as a functional trait. This creates a systematic disconnect between laboratory screening and effective rhizosphere establishment under field conditions. (B) In real soil environments, inoculated strains must navigate tortuous pore networks, discontinuous water films, and dead-end pores while sensing and responding to dynamic chemical gradients generated by root exudates. These combined physical and chemical constraints, operating at the mm-to-cm scale, constitute a motility-dependent colonization bottleneck. (C) Under field conditions, this bottleneck manifests as variable crop growth responses and unstable rhizosphere colonization outcomes. (D) Spatiotemporal dynamics further influence colonization success. Diurnal physiological changes, irrigation events, soil moisture fluctuations, and plant developmental stages jointly define a brief but critical colonization window, within which motility-mediated chemotactic orientation becomes decisive for competitive root establishment. Figure created with Biorender.com.

In this perspective article, we argue that motility is an important yet underappreciated determinant of the field performance and stability of microbial inoculants in the soil environment, and we outline the major motility strategies and their coupling to plant-derived chemical signals, situating them within the broader framework of microbial ecology, particularly microbial dispersal and biotic interactions in the rhizosphere [12]. We further discuss the ecological advantages conferred by microbial motility during microbial inoculant application, including spatial accessibility, interactions with resident microbial communities, competitive positioning, priority effects during colonization, and long-term persistence, as well as the trade-offs between motility and stable root colonization. We propose a multi-level engineering framework for systematically incorporating motility into biofertilizer development pipelines, where “engineering” refers to the rational, design-driven integration of motility traits rather than genetic modification alone, encompassing strain screening, consortium design, regulatory tuning, and deployment strategies, ultimately improving the stability and predictability of microbial inoculant performance under field conditions.

## Microbial motility in the rhizosphere

Recent advances in cryo-electron microscopy and fluorescence-based tracking techniques have elucidated the structural bases of flagellar rotation, pili extension and retraction, and gliding motility [16], as well as the signalling pathways that govern chemotaxis, from stimulus perception and signal transduction to motor output [17]. These studies have revealed how bacteria sense and respond to chemical gradients to gain ecological competitive advantages [18] (Table 1). However, current understanding of bacterial motility is largely derived from a small number of model strains such as *Escherichia coli* under well-mixed liquid-phase conditions with relatively stable chemical gradients [19]. In such idealized environments, *E. coli* achieves swimming velocities of roughly 20–35 μm s^−1^ through flagellar rotation [20] and migrates directionally along chemoattractant gradients using a run-and-tumble motility strategy [16]. At this rate, traveling 1 cm would require ~5–8 min of continuous, unidirectional swimming under ideal laboratory conditions. By contrast, in a soil-based olfactometer assay in which six soil bacterial isolates spanning the genera *Burkholderia, Collimonas, Dyella, Janthinobacterium, Paenibacillus, and Pseudomonas* migrated towards volatiles emitted by *Carex arenaria* roots, a significant increase in bacterial abundance relative to the no-attractant control was detected only after 65 h of incubation at a sampling point located 3.8 cm from the inoculation site [21]. These observations derive from different organisms and experimental systems and therefore do not quantify how much slower migration is in soil. The striking difference in timescale is nonetheless consistent with the physical constraints detailed below, which impose far greater limits on bacterial movement in structured soil than under idealized liquid conditions.

**Table 1:** Molecular mechanisms and ecological relevance of bacterial motility modes in the rhizosphere.

Motility type	Molecular machinery	Typical speed	Environmental requirement	Key regulatory signal	Ecological relevance in the rhizosphere	Key references
Swimming	Rotating helical flagella (~20 nm diameter); chemotaxis via CheA/CheY two-component system; run-and-tumble navigation	~20–100 μm s^−1^	Continuous water films in soil pores	Phosphorylated CheY modulates flagellar motor switching	Enables chemotactic navigation toward root exudate gradients; can traverse a 1 mm soil pore in under 1 min	[12, 16, 83]
Swarming	Hyperflagellation; surfactant and osmoprotectant secretion; collective movement-generating vortices	~0.1–10 μm s^−1^	Moist nutrient-rich surfaces	Surface contact triggers mechanosensory signalling; c-di-GMP modulation; quorum sensing at population level	Facilitates rapid colonization of mucilage-covered root surfaces; collective behaviour amplifies colonization efficiency	[12, 31, 71, 108]
Twitching	Type IV pili extension and retraction; polar pili localization regulated by mechanical input	~0.1–1 μm s^−1^	Solid surfaces or hydrated interfaces	Pil-Chp chemosensory system integrates mechanical signals; elevated c-di-GMP promotes biofilm transition upon surface contact	Critical for precise root surface positioning and endophytic invasion; pilT/pilA mutants show severe colonization defects in multiple PGPRs	[16, 109–111]
Gliding	Surface adhesin (e.g. SprB) movement along helical cell envelope tracks; independent of flagella and pili	~2–4 μm s^−1^ (Flavobacterium); ~2–5 μm min^−1^ (Myxobacteria)^−1^	Surface-associated environments with limited water films	No direct coupling to classical chemotaxis; specific regulatory signals unclear	Potential advantage in drier rhizosphere microhabitats; contribution to root colonization remains poorly characterized	[16, 33, 112]

Rhizosphere soils are generally unsaturated, resulting in the fragmentation of continuous water films into isolated microdroplets, severely constraining long-distance migration that depends on flagellar swimming [14]. Moreover, the rhizosphere pore network is highly tortuous. Geometric tortuosity measurements based on X-ray tomography of intact soil aggregates indicate that migration paths are typically ~1.2–2.0 times longer than the corresponding Euclidean distance, thereby increasing the distance that bacteria must travel to reach their targets [22]. The presence of dead-end pathways in soil can trap bacterial cells within pore spaces, further reducing chemotactic efficiency [23–25]. Plant roots are themselves dynamic rather than static targets, continuing to grow over timescales of days and progressively reshaping the surrounding soil environment both horizontally and vertically, thereby changing the spatial separation between roots and bacterial populations. Hence, once passive dispersal and continued root growth bring bacterial cells into the vicinity of the root, bacterial motility becomes a key factor facilitating the final short-range approach to the root surface.

In addition to physical constraints, the chemical landscape of the rhizosphere is intrinsically complex. Plant roots simultaneously secrete multiple classes of compounds, including sugars, organic acids, amino acids, and secondary metabolites, with concentrations varying substantially not only over short time scales but also with root age and spatial position along the root system. The composition of root exudate differs markedly between actively growing root tips and more mature root segments, especially for secondary metabolites [26, 27]. This chemical environment is further shaped by the resident microbiota, whose metabolites, including volatile compounds, add to the cues available to incoming bacteria and can modulate their motility and behaviour in soil [28]. Therefore, chemotaxis models based solely on single-compound concentration gradients are insufficient to explain bacterial migration under rhizosphere conditions [29, 30]. Whether these conceptual frameworks can be directly extended to the rhizosphere remains a fundamental open question.

To cope with the spatial and temporal heterogeneity of the rhizosphere, microbes rely on active dispersal through distinct modes of motility, including swimming, swarming, twitching, and gliding, each adapted to particular microenvironmental conditions (Table 1). Swimming, driven by flagellar rotation, is the fastest form of bacterial motility in liquid environments and rapidly responds to gradients of root exudates [12, 16]. As water films become fragmented under these conditions, swimming gives way to surface-associated modes such as swarming. Swarming is a collective behaviour that occurs on semi-solid surfaces and has been observed on media containing ~0.3%–1% agar [31]. During swarming, bacteria maintain local water films and achieve rapid population-level expansion through the secretion of osmolytes and biosurfactants [16]. In drier microenvironments or habitats dominated by solid surfaces, twitching motility mediated by type IV pili becomes critical. Through cycles of pilus extension, adhesion, and retraction, twitching enables precise positioning on root hair surfaces, thereby providing a foundation for subsequent microcolony formation and biofilm development [32]. In addition, some rhizosphere bacteria, such as *Flavobacterium johnsoniae*, possess gliding motility, which relies on the movement of adhesin proteins along the cell surface [33]. These motility modes coexist in some bacterial strains and can be dynamically switched on in response to environmental cues, thereby enabling continuous adaptation across spatial gradients in the rhizosphere [12, 34]. For example, the rhizosphere-associated diazotroph *Azospirillum brasilense* switches from swimming to swarming upon surface contact, facilitating attachment to and colonization of wheat roots [35]. Despite the growing recognition of the ecological relevance of motility mode diversity, empirical evidence on the environmental conditions under which different motility modes are beneficial remains limited, as most knowledge derives from laboratory studies of model organisms [36].

## Why microbial motility matters for biofertilizer success?

### Motility and spatial access to the root surface

The colonization efficiency of bacterial inoculants in biofertilizers depends in part on their ability to actively move through structurally complex soil pore networks and access the root surface [16, 37, 38]. A substantial body of genetic evidence demonstrates that flagellar motility plays an important role in this process. For example, in competitive colonization assays using *Pseudomonas fluorescens* F113, when flagellar mutant strains and the corresponding wild type were co-inoculated, nonmotile mutants carrying disruptions in flagellar genes (*fliC, fliS, fleQ*) exhibited severely impaired rhizosphere colonization, although these mutants showed normal growth in liquid culture. Impaired rhizosphere colonization led to progressive exclusion from the root-associated niche by the wild-type strains during colonization [39]. Similarly, nonmotile mutants of *Bacillus subtilis* lacking flagellar function (Δ*hag* or Δ*motA*) were almost completely unable to establish effective colonization on *Arabidopsis thaliana* roots, indicating that flagellum-mediated motility is essential for the initial physical encounter between bacterial cells and the root surface [40]. Quantitative analyses in tomato further revealed that the colonization efficiency of a nonflagellated *B. subtilis* mutant (Δ*hag*) was reduced to only 0.47% of that of the wild-type strain [41]. This dependence is also reflected in some widely adopted microbial inoculants. *Azospirillum brasilense*, one of the most extensively studied and commercially deployed plant growth-promoting (PGP) bacteria, exhibits flagellum-driven motility and chemotaxis that enhance root colonization and increase the probability of root–bacterial interactions [35, 42, 43]. Recent inoculant studies further show that enhancing chemotactic recruitment through root exudate supplementation improves rhizosphere colonization and plant growth, in one case increasing seed colonization by about three orders of magnitude [44], highlighting motility as a practical determinant of inoculant performance rather than merely a laboratory phenotype. Although the genetic evidence derives largely from controlled laboratory and gnotobiotic systems, it collectively suggests that motility may substantially influence colonization success alongside metabolic or PGP traits. This raises the possibility that strain selection strategies based primarily on metabolic functions or PGP traits may be insufficient, as strains with strong functional potential under laboratory conditions may still fail to establish rhizosphere populations at ecologically relevant densities if they are unable to effectively access the root surface (Fig. 1C and D). Whether this motility-dependent colonization process translates directly to field soils, where resident microbiota, heterogeneous pore structures, and fluctuating hydration conditions impose additional constraints, remains to be tested directly.

### Motility, priority effects, and competitive positioning during colonization

Beyond spatial accessibility, motile bacteria may also gain a transient first colonizer advantage when competing for newly formed root surfaces. Early-arriving microbes can modify local microenvironmental conditions in ways that enhance their own adaptation and long-term persistence, confer an advantage over later arrivals through the pre-emption of attachment sites and nutrient resources and the alteration of local physicochemical conditions, and influence which later-arriving taxa successfully establish, thereby shaping the subsequent trajectory of community assembly on the root. These arrival-order-dependent effects are collectively referred to as priority effects [45, 46]. For introduced inoculants, however, opportunities to exploit such priority effects are constrained because they enter an ecosystem in which resident microbial communities are already established. Emerging roots may also be colonized by seed-borne microbes transmitted with the seed, which, together with soil residents, can contribute to early root colonization before an introduced strain arrives [47]. However, the composition and diversity of seed microbiota vary substantially among plant species and among individual seed samples, indicating that the potential contribution of seed-borne microbes to early root colonization is unlikely to be uniform across plant systems [48]. Because these prior colonizers may occupy developing root surfaces early, an introduced strain must reach the root sufficiently rapidly to compete for available niches. This process can begin with passive dispersal, which transports cells towards the root zone and may strongly influence whether they reach the vicinity of the developing root. Once within this local environment, motility can facilitate directed movement towards the root surface, thereby influencing arrival timing and the opportunity for subsequent competitive establishment. However, root systems grow continuously, generating fresh root surfaces and newly released exudate hotspots. In some systems, motility is particularly influential at the youngest root zones, where chemotactic strains such as *B. subtilis* and *P. fluorescens* preferentially and competitively colonize the root tip and elongation zone [49, 50]. At the community scale, however, these same zones have been reported to be among the least altered following inoculation, whereas more mature root regions are more strongly affected [51]. Because these observations characterize colonization at different scales, whether rapid, motility-driven arrival confers a genuine advantage on newly formed surfaces remains an open question. The ability of an introduced strain to arrive rapidly may nonetheless confer a localized colonization advantage, and the timing of inoculation has indeed been proposed as a determinant of inoculant establishment [52, 53]. Direct evidence on whether such fine-scale priority advantages translate into improved biofertilizer performance under field conditions, however, remains scarce. During biofertilizer application, motile bacteria can rapidly sense and respond to newly established, steep chemical gradients generated by root exudates via chemotaxis, thereby gaining a competitive advantage over nonmotile counterparts. This creates a brief yet critical opportunity window allowing motile bacteria to reach the root surface, initiate attachment, and begin biofilm formation ahead of nonmotile competitors [9, 40, 42, 54] (Fig. 2A).

**Figure 2:**
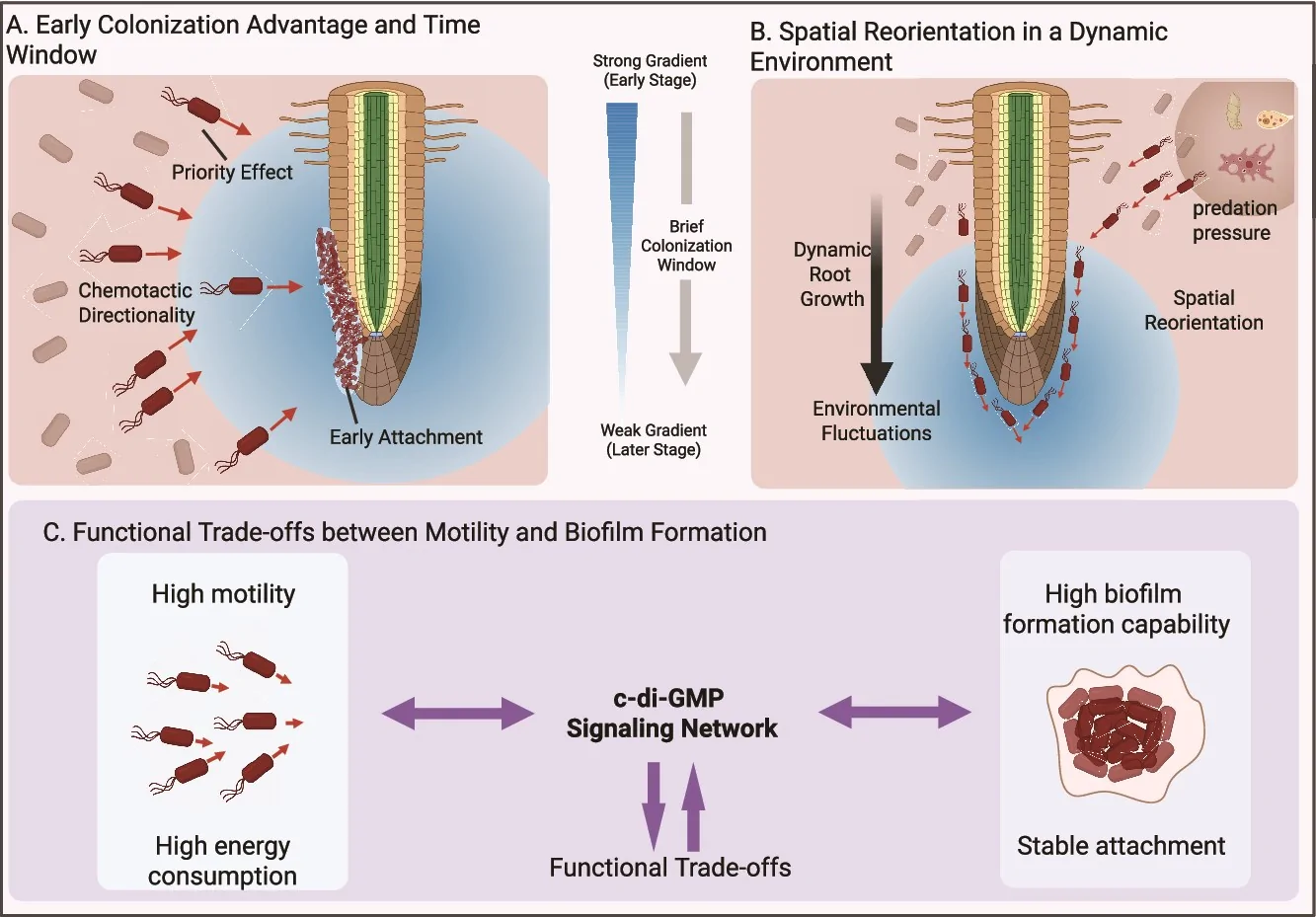
Motility-dependent ecological mechanisms and trade-offs during rhizosphere colonization. (A) Motility confers a pioneer advantage during the early stages of rhizosphere colonization. Strains capable of rapidly sensing and responding to newly established chemical gradients generated by root exudates through chemotaxis can preferentially reach the root surface within the period when gradients are steepest and signals are strongest. Upon arrival, they occupy limited attachment sites and initiate early biofilm formation. This early arrival not only enhances the competitive positioning of individual strains but also modifies local microenvironmental conditions, thereby generating priority effects that influence subsequent microbial community assembly. (B) In dynamically changing soil environments, motility further enables continuous spatial repositioning. As roots extend at millimetre-scale rates and alter their exudation patterns, and as soil moisture and pore connectivity fluctuate, motile bacteria can continuously track newly formed root tips and actively avoid localized stressors. This capacity allows them to maintain persistence within favourable microhabitats. In contrast, nonmotile strains are more likely to remain confined to regions experiencing resource depletion or increasing environmental stress. (C) Although motility provides significant advantages in early competition and environmental adaptation, its ecological value is constrained by energetic costs and regulatory control. The synthesis and continuous rotation of flagella require substantial energy investment. In addition, the c-di-GMP signalling network, which promotes cell attachment and extracellular polymeric substance production, typically suppresses the expression of motility-related genes, thereby establishing a functional trade-off between motility and biofilm formation. Figure created with Biorender.com.

Beyond conferring an advantage during early colonization, bacterial motility may also influence patterns of competition and coexistence within rhizosphere microbial communities at a broader ecological scale. In soil environments, strains characterized by different motility and growth trade-offs often spontaneously form spatially segregated distributions during colonization. Through active spatial segregation within nutrient patches, each strain can gain a competitive advantage when rare, producing an inversion of the competitive hierarchy that promotes stable coexistence among strains [55]. Once a stable microbial community is established in the rhizosphere, early colonizers can monopolize limited attachment sites, preferentially exploit local nutrient pools, and in some cases, secrete antimicrobial compounds that inhibit late arriving strains, further reinforcing their ecological advantage [56–58]. Consequently, successful establishment of newly arriving bacteria increasingly depends on their ability to compete with resident populations and integrate into pre-existing biofilms, processes that can be facilitated by ecological traits such as secretion systems and biofilm-modifying enzymes [59, 60]. The timing of colonization is critical because the release of root exudates can reach saturation or shift significantly within hours, and some compounds display clear diurnal patterns [61]. Taken together, these findings suggest that, for biofertilizers applied during sowing and early plant growth stages, the capacity of microbial inoculants for rapid chemotactic response and timely tracking of rhizosphere signals is critical for root colonization.

### Motility and long-term persistence under dynamic conditions

Besides facilitating initial root encounter, bacterial motility fulfils an important but often overlooked function by enabling spatial repositioning in response to changing environmental conditions [9, 62]. Plant roots grow at rates of millimetres per day, generating new root surfaces, whereas older root regions progressively enter stages of secondary development accompanied by reduced exudation [63, 64] (Fig. 2B). Soil moisture and pore connectivity also fluctuate in response to irrigation practices and wet–dry cycles. Under such highly dynamic conditions, motile bacteria must flexibly switch between different motility modes in accordance with changing environmental cues to maintain fitness [65], whereas nonmotile bacteria are more likely to become trapped within progressively deteriorating microsites and are unable to migrate to newly favourable regions [66]. Because actively motile bacteria are unlikely to keep pace with the rapid elongation of crop roots under unsaturated soil conditions, bacterial motility is better viewed as the active, short-range component of dispersal that enables repositioning towards newly favourable microsites and escape from localized stressors such as predator aggregations [67]. Over longer spatial scales, redistribution that maintains proximity to growing roots is likely to depend more strongly on passive dispersal by water flow, fungal hyphae, and soil fauna [34, 68, 69]. We hypothesize that, compared with strains possessing comparable metabolic potential but limited motility, motile strains may maintain more stable rhizosphere populations throughout the growing season, whereas poorly motile strains may be more susceptible to population fluctuations driven by local environmental changes. However, direct empirical validation of this hypothesis in rhizosphere systems warrants further investigation. The persistence of biofertilizers under field conditions may depend largely on the ability of bacterial cells to maintain and regulate motility after initial colonization, but this critical behavioural trait has been almost entirely overlooked in current strain screening frameworks.

### Trade-offs between motility and biofilm-mediated persistence

Despite the advantages outlined above, bacterial motility is associated with substantial costs (e.g. the biosynthesis and sustained rotation of flagella) that must be balanced against its potential benefits. For an individual bacterium, motility does not confer a universal adaptive advantage, and its ecological value is strongly context dependent. In resource-rich microsites, such as regions influenced by root exudates, the energetic cost of flagellar construction and operation may account for a small fraction of the total cellular energy budget; however, this investment can rise dramatically, in some cases approaching several tens of percent of the total cellular energy expenditure, particularly in small cells or species with extensive flagellar apparatuses [70]. Within a given bacterial population, motility and biofilm formation can represent alternative phenotypic states that are dynamically regulated in response to environmental conditions, introducing an additional layer of complexity to rhizosphere colonization. This switch between motile and sessile states can be governed by diverse regulatory systems, among which bis-(3′–5′)-cyclic dimeric guanosine monophosphate (c-di-GMP) signalling is most extensively characterized. c-di-GMP promotes cell-surface attachment and extracellular polysaccharide production while typically repressing flagellar gene expression [71, 72] (Fig. 2C). Other regulatory mechanisms include bistable transcriptional circuits, such as the SinR-SlrR-SinI switch in *B. subtilis* [73], as well as small-RNA-mediated regulation and quorum-sensing inputs [74, 75]. Together, these regulatory systems mediate a trade-off between two capacities required at different stages of root colonization: motility facilitates access to the root surface, whereas biofilm formation supports stable attachment and persistence once the surface has been reached. Because bacterial populations can shift dynamically between motile and sessile states, this trade-off need not represent a fixed constraint but can instead be accommodated by balancing these functional demands at different stages of colonization.

We argue that the trade-offs described above are better interpreted as interchangeable phases in the cyclic development of bacterial populations rather than as a permanent, one-way constraint on bacterial physiology. During the critical window of initial colonization, increased motility is essential for achieving spatial accessibility, chemotactic orientation, and competitive positioning. Subsequently, a programmed reduction in motility accompanied by enhanced biofilm formation may be more favourable for long-term persistence in the rhizosphere [76] (Fig. 3A). As biofilms mature, they can in turn disperse and release motile cells, a process that can be influenced by quorum sensing, allowing bacterial populations to reposition and colonize newly favourable microsites; these transient transitions may therefore be ecologically important for both persistence and repositioning [77]. Bacterial strains that can transition between motile and sessile states in response to local cues, such as surface contact, quorum sensing signals, interspecific signalling, and other microenvironmental stimuli, may therefore represent promising candidates for biofertilizer development [78–80]. In addition, the ecological advantages conferred by motility are strongly dependent on the specific deployment context. In soils with high moisture availability, recent tillage, and high pore connectivity, flagellum-mediated swimming may be decisive for effective rhizosphere access (Fig. 3B). In contrast, under soil compaction or drought, passive dispersal mechanisms, including hitchhiking via biological vectors such as fungal hyphae and soil fauna, may become increasingly important for long-distance redistribution of bacterial cells, whereas type IV pili–dependent twitching motility facilitates short-range movement among neighbouring microsites [68, 69, 81] (Fig. 3B). Therefore, motility related traits should be contextually matched and optimized during biofertilizer development, according to the physical characteristics and management conditions of the target agricultural environment. Once passive dispersal has brought cells close enough to the root, motility enables short-range movement towards the root surface. Subsequent processes, including competition with resident microbial communities and establishment within pre-existing biofilms on the root surface, population dynamics within the rhizosphere, and the stable expression of plant-beneficial functional traits, jointly determine the overall field performance of microbial inoculants.

**Figure 3:**
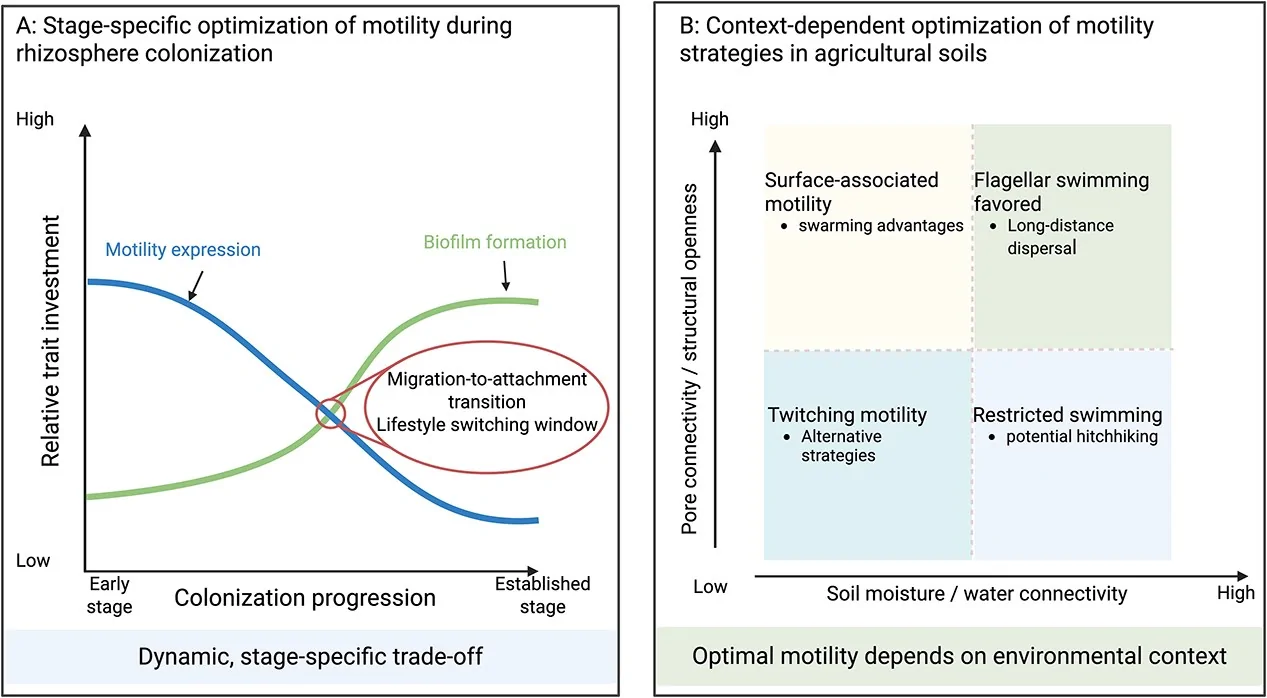
Stage-specific trade-offs and context-dependent optimization of bacterial motility during rhizosphere colonization. (A) Motility should be interpreted as a dynamic, stage-structured trait rather than a fixed physiological advantage. During early colonization, elevated motility facilitates spatial access, chemotactic orientation, and competitive positioning. As colonization progresses, a transition towards biofilm formation becomes favourable for stable rhizosphere persistence. The intersection of these trajectories represents a migration-to-attachment transition window, highlighting the temporal rebalancing between dispersal and stable establishment. (B) The ecological benefit of motility is strongly dependent on environmental context. Optimal motility strategies mainly vary with soil moisture and pore connectivity. Flagellar swimming is favoured in well-connected, water-saturated soils that permit long-distance dispersal, whereas surface-associated motility or twitching may confer advantages under limited moisture or restricted pore connectivity. Figure created with Biorender.com.

## Engineering motility into next-generation biofertilizers

In conventional biofertilizer development frameworks, motility has rarely been considered as a core design and screening parameter for microbial inoculants [13, 82]. Here, we propose a systematic, multi-level framework of several stages to incorporate motility into biofertilizer screening criteria, microbial consortium design, regulatory strategies, and field deployment strategies (Fig. 4).

**Figure 4:**
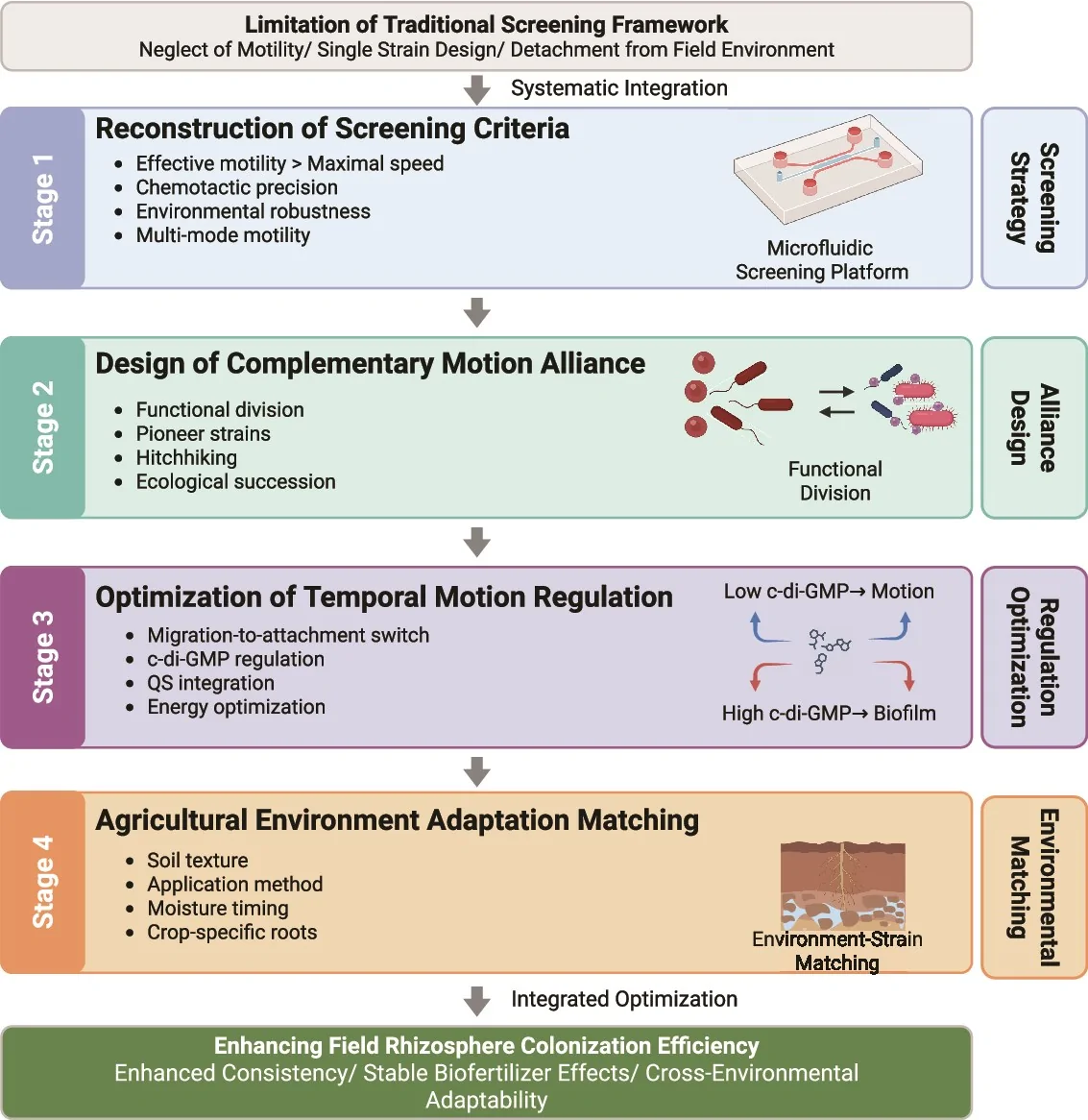
A multi-level engineering framework for integrating bacterial motility into biofertilizer development. This framework outlines a staged strategy for systematically incorporating bacterial motility into the full biofertilizer development pipeline, from strain screening to field deployment. Stage 1: Reconstruction of screening criteria. Screening strategies shift from measuring maximal swimming speed under ideal laboratory conditions to evaluating ecologically effective motility. Key parameters include chemotactic precision, environmental robustness, stress-recovery capacity, and functional diversity across motility modes, assessed using platforms such as microfluidic systems and transparent soil models. Stage 2: Design of motility-complementary consortia. Motility is treated as a collective trait distributed across consortium members. Functional division between pioneer strains and performance-oriented strains, along with complementary migration modes, enhances spatial coverage and increases colonization probability in heterogeneous soil environments. Stage 3: Optimization of temporal motility regulation. Efficient rhizosphere establishment requires a controlled transition from migratory to sessile lifestyles. Regulatory hubs such as c-di-GMP signaling and quorum sensing networks can be leveraged to coordinate motility expression with biofilm formation, balancing early dispersal efficiency with long-term stability. Stage 4: Agricultural environment adaptation matching. The ecological benefits of motility are context-dependent and must be aligned with soil texture, application strategy, moisture timing, and crop-specific root traits. Matching strain-level motility characteristics with field conditions is essential for translating laboratory performance into consistent rhizosphere establishment. Collectively, these four levels of integration aim to enhance the consistency, stability, and cross-environment adaptability of biofertilizer performance under field conditions. Figure created with Biorender.com.

### Stage 1. Incorporating microbial motility into microbial inoculant screening strategies

Bacterial motility is commonly simplified as swimming speed measured under idealized experimental conditions [31]. However, quantitative metrics derived from these assays primarily reflect the maximal motility potential of bacteria, rather than their functional capacity to reach and colonize the rhizosphere [31, 83]. If motility is to be treated as a design variable in microbiome engineering, screening approaches should move beyond assessing whether motility is present to evaluating whether motility is ecologically effective. Ecologically meaningful metrics should capture the precision of bacterial chemotactic responses to chemical gradients generated by root exudates [18, 26], namely, the ability to detect, integrate, and directionally navigate complex chemical landscapes [19]. In addition, as temperature and resource availability in soil environments are inherently dynamic, screening criteria should assess whether bacteria can sustain stable motility across realistic ranges of temperature and moisture, as well as under carbon or nutrient limited conditions [14].

Current screening of bacterial motility primarily relies on plate-based motility assays together with endpoint assessments of root colonization and therefore provides only static measurements at a single time point [31]. Consequently, these approaches fail to capture the dynamic transitions between motile and sessile states in response to environmental stress. In soil, cyclical fluctuations in water availability imply that bacterial motility is not continuously expressed; under unfavourable conditions, transition towards a biofilm state may provide protection, whereas motility can be reinitiated once favourable conditions are restored [12, 83, 84]. Strains capable of dynamically switching between these states and rapidly resuming motility after stress relief may therefore have a competitive advantage in sustaining colonization during rhizosphere expansion. Finally, attention should be paid to fundamentally different ecological advantages among distinct modes of movement, such as swimming, collective surface spreading, and type IV pili–mediated twitching motility (Table 1) [12, 31, 34]. Screening frameworks that can compare combinations of motility modes, rather than focusing on a single form of movement, are more likely to identify strains with effective colonization strategies. These considerations also define how far genomic screening alone can go. Genome sequences readily reveal the presence and diversity of motility and chemotaxis machinery, including flagellar, type IV pili and gliding genes, and the size of the chemoreceptor repertoire, and are therefore a useful first filter; gene content, however, indicates capacity rather than realized behaviour, and whether movement is effective in structured, unsaturated soil cannot be inferred from sequence alone. The absence of flagellar genes should therefore not by itself disqualify a candidate because twitching and gliding can sustain movement precisely where flagellar swimming is least effective, and weakly motile strains can still reach roots through passive dispersal, hitchhiking on motile or fungal partners, or near-root placement, and can serve as functionally potent members of a consortium. Motility is thus best applied as a graded, context-weighted criterion rather than a binary filter.

A range of emerging platforms may help enable such shift in screening criteria. For example, microfluidic devices allow real-time tracking of bacterial chemotactic responses to defined chemical gradients at single-cell resolution [49, 85, 86]. Transparent soil systems, constructed from Nafion particles combined with refractive index–matched solutions, permit 3D imaging of bacterial distributions within structurally realistic porous media [87, 88]_._ Controlled plant–soil systems can be used to assess the ability of candidate strains to traverse soil matrices and reach the root surface, thereby integrating motility with other traits relevant to early colonization. These systems can also yield quantitative ecological data on the effective spatial range of active bacterial motility and on its contribution relative to passive dispersal during migration and rhizosphere colonization. The high-dimensional, multi-modal datasets generated by these platforms are well suited to machine-learning approaches that can be used to integrate motility phenotypes with soil, crop, and management variables and develop predictive models of rhizosphere colonization under field conditions.

### Stage 2. Engineering motility-complementary consortia for biofertilizer design

A single bacterial strain rarely achieves optimal performance across multiple dimensions, including spatial migration, stable colonization, and functional expression [10]. This limitation is particularly pronounced under field conditions, where trade-offs often emerge among high motility, strong PGP functions, tolerance to diverse abiotic stresses, and competitiveness in biotic interactions with the resident microbiota [10]. In light of these constraints, biofertilizer design should treat motility as a collective trait that can be functionally partitioned among consortium members to optimize overall performance [89]. Several mechanisms support this concept, among which microbial hitchhiking is one of the most illustrative examples [68, 90, 91]. Recent studies have demonstrated that non-motile bacteria can achieve long range spatial dispersal by attaching to the flagella of motile partners and exploiting their motility machinery [68, 92]. For instance, *Streptomyces* spores can adhere to the flagella of *B. subtilis* or *P. fluorescens*, enabling centimetre-scale migration towards plant roots. This phenomenon provides a conceptual blueprint for the rational design of next-generation biofertilizers, in which highly motile but functionally limited bacterial strains represent one class of biological carriers that facilitate the transport of functionally potent yet weakly motile cargo strains through physical or ecological interactions, thereby achieving synergistic colonization [68]. Similar design principles may also be extended to fungal partners. Fungal mycelia can act as “fungal highways,” providing continuous water films along which motile bacteria can disperse at rates of up to ~1 cm per day [69, 93]. This mechanism is directly relevant to the rhizosphere: the bacterial endophyte *Rahnella aquatilis* exploits the directed hyphal growth of *Fusarium oxysporum* to reach and colonize plant roots [94].

Microbial consortia can further extend their activity range through complementary motility modes. Soil is characterized by heterogeneous moisture states, pore size distributions, and surface properties, where distinct motility mechanisms confer different advantages [12, 25]. Swimming facilitates migration through water filled pores, collective surface spreading is favoured on moist surfaces, and type IV pili–mediated twitching is better suited for movement through narrow channels or under low-moisture conditions [31, 81, 83]. By assembling strains that exhibit different motility modes, consortia can collectively occupy environmental niches that are inaccessible to any single strain, thereby increasing the overall probability of migration and colonization in soils. Complementarity is not limited to movement. In some cases, co-occurring species produce more biofilm together than any of the component species does alone, and thus, consortium composition can influence not only how far cells travel but also how stably they attach once the root surface is reached [95]. Consortium design can also introduce functional partitioning across temporal scales. Highly motile strains may act as pioneers that reach the rhizosphere early and modify the local microenvironment, for example, by outcompeting resident microbes, releasing signalling molecules, or establishing initial biofilms, thereby creating more favorable conditions for subsequent colonizers [45, 58]. Because these emerging surfaces may already be colonized by resident microbiota, an effective pioneer must not only arrive rapidly but also compete successfully with established populations; selecting strains that combine motility with competitive and invasion-related traits, such as type VI secretion systems, antimicrobial production, and mechanisms that facilitate invasion into or integration within pre-existing biofilms, is therefore a promising design criterion [59, 60]. Thereafter, strains with weaker motility but stronger PGP functions can persist and deliver their benefits within the niche established by the pioneers. This relay-like division of labour offers a conceptual framework for biofertilizer design but remains challenging in practice because it requires careful consideration of strain compatibility, the balance between competition and cooperation, the potential for both mutualistic and pathogenic interactions, and the maintenance of stability during large-scale production.

### Stage 3. Manipulating microbial motility for effective rhizosphere colonization

Once candidate strains with effective motility are identified during screening, successful rhizosphere colonization ultimately depends on whether motility can be expressed at appropriate temporal and spatial scales [65]. For biofertilizer applications, continuous expression of high levels of motility does not represent an optimal state for rhizosphere establishment [70]. Flagellar synthesis and operation impose substantial energetic costs, and strains that maintain prolonged high motility often fail to activate the molecular programs required for stable biofilm development [72, 96]. Consequently, efficient and persistent rhizosphere colonization relies on an effective transition from a migratory to a sessile lifestyle [9]. In this process, the transition between motile and sessile states is controlled by diverse bacterial motility–sessility switch systems, among which c-di-GMP signalling is one of the most extensively characterized examples [97]. Low intracellular levels of c-di-GMP promote the expression of flagellar-associated genes and sustain motile behaviour, whereas accumulation of c-di-GMP suppresses motility while activating extracellular matrix production and biofilm formation. This regulatory logic is highly conserved across diverse bacterial taxa, making it an attractive target for engineering-based intervention [98]. Two opposing enzyme families set c-di-GMP levels and constitute the main entry points. Diguanylate cyclases, which carry GGDEF domains, synthesize c-di-GMP, whereas phosphodiesterases, which carry EAL or HD-GYP domains, degrade it [72]. By modulating the activity or expression of these enzymes, it may be possible to reprogram the timing and dynamics of motility expression without altering core metabolic functions [99]. Such interventions should nonetheless be applied with caution, as biofilm-related gene expression is highly sensitive to environmental cues, including interspecies interactions that can themselves reshape biofilm formation [80]. Engineering outcomes are therefore likely to be context-dependent. This context dependence represents a practical barrier to commercialization because inoculant producers generally seek broadly applicable solutions that perform consistently across diverse soils and crops. A realistic strategy may therefore be to define the range of soil, moisture, and application conditions under which a motility-engineered strain is expected to perform reliably.

Temporal control of motility can be achieved through multiple engineering strategies. For example, enhanced expression of phosphodiesterases can maintain low intracellular levels of c-di-GMP, thereby extending the motility window and providing strains with additional time to reach the rhizosphere [72]. However, such manipulation should preserve the capacity to subsequently transition to a sessile state, allowing stable attachment and biofilm formation once the root surface has been reached. An alternative strategy involves coupling motility regulation to quorum-sensing systems, such that bacteria remain motile at low population density but automatically suppress motility and initiate biofilm formation upon aggregation at the root surface [79]. Recent advances in synthetic biology suggest that modulation of promoter strength for flagellin genes can also alter flagellar expression levels and thereby regulate bacterial motility behaviour [100]. However, enhancing bacterial motility in plant-associated environments may also introduce additional ecological constraints. Flagellar components, particularly conserved flagellin epitopes such as flg22, can be recognized by plant pattern recognition receptors and activate innate immune responses. As a result, highly motile bacteria approaching the root surface may face an increased risk of immune recognition, which could, in turn, reduce colonization efficiency [101]. This cost, however, is not fixed. Flg22 epitopes vary widely among plant-associated bacteria, and most commensal variants evade recognition, whereas others modulate immune signalling [102]. In some taxa, distinct flagellins are functionally specialized for swimming and for host colonization, such that the immune receptor surveils the variant required for colonization rather than the one required for swimming [103]. Therefore, engineering strategies aimed at regulating bacterial motility should also take these potential ecological constraints into account, including the immunogenicity of the motility structures themselves, to balance motility performance, colonization efficiency, and host immune responses. Although the practical application of genetically regulated motility control strategies in agricultural systems still faces challenges related to stability and regulatory acceptance, a deeper understanding of these regulatory mechanisms can nevertheless provide an important theoretical basis for strain selection and microbial consortium design.

### Stage 4. Matching microbial motility traits to agricultural contexts

Effective field deployment requires the alignment of strain-specific motility traits with application methods and prevailing field conditions. First, soils with contrasting textures differ markedly in pore size distribution and connectivity, imposing distinct constraints on bacterial movement [84]. Sandy soils typically possess larger pores and higher water connectivity and, under sufficiently moist conditions, favour flagellum-driven swimming and passive water-mediated dispersal [14]. In contrast, clay-rich soils are characterized by small pore sizes and low connectivity, where surface associated twitching motility or other nonswimming migration strategies confer greater advantages [84]. Soil compaction can further reduce pore size and connectivity, limiting swimming and water-mediated dispersal even in coarse-textured soils [15, 84]. Second, application method further determines the functional requirement for motility, by defining the initial spatial positioning of inoculated bacteria. Seed coating places bacteria directly adjacent to emerging roots, substantially reducing the distance required for migration, such that even strains with limited motility may successfully establish colonization [104]. In contrast, furrow application or soil broadcasting requires bacteria to traverse millimetre to centimetre scale distances through soil, imposing greater demands on both motility and chemotactic navigation [12]. Accordingly, application methods should place inoculants sufficiently close to the target root zone, while agricultural practices that facilitate passive dispersal can support longer-distance transport, leaving active motility to mediate the final approach to the root. Consequently, highly motile strains are likely to be most advantageous when bacterial cells must still actively bridge a short distance, whereas strains with weaker motility but stronger functional traits may perform better under direct near-root placement strategies such as seed coating.

Third, soil moisture at the time of inoculation represents a critical determinant of whether motility can be effectively expressed. Bacterial migration depends on the presence of continuous water films, and under favourable moisture conditions, pore scale water connectivity is maintained, allowing motile strains to gain an advantage in reaching the rhizosphere [83]. As soils dry, pore spaces become increasingly disconnected, bacterial migration pathways are fragmented, and the benefits conferred by motility rapidly diminish [84]. Coordinating inoculation timing with irrigation or rainfall events during sowing or biofertilizer application can therefore promote water-mediated passive dispersal while maintaining the water connectivity required for active motility during early colonization. More broadly, agricultural practices that maintain soil organic matter and support soil fauna may provide additional opportunities for passive bacterial dispersal, complementing active motility during inoculant establishment [105]. Finally, crop-specific root traits also influence the suitability of motility-based strategies. Differences in root system architecture and exudate composition shape the spatial distribution and effective range of rhizosphere signals [106]. Crops with dense and shallow root systems may more readily intercept inoculants applied near the soil surface, whereas deep rooted crops require strains with more persistent motility or higher chemotactic precision. Beyond matching strains to field conditions, formulation design offers another avenue for improving inoculant deployment. Additives that maintain local hydration or reduce surface tension, such as humectants and surfactants, may help maintain the water connectivity required for active motility and improve bacterial dispersal through soil [14, 107]. Whether formulations that promote controlled cell aggregation could further improve inoculant delivery remains unclear because aggregation may not only facilitate transport with percolating water under some conditions but also increase straining and retention within fine pore networks [25, 84]. Together, these considerations indicate that motility should not be treated as a universally optimal design target but rather as a context-dependent trait to be optimized alongside application and formulation strategies within specific crop and soil systems. This context dependence is also an important consideration for the development and deployment of effective biofertilizer solutions, as context-specific products entail different requirements for testing, validation, and implementation than broadly applicable solutions.

## Concluding remarks

Motility is a critical but neglected trait in strain screening pipelines and biofertilizer development. It influences whether inoculated bacteria can physically reach the rhizosphere once delivered into its vicinity, compete for root surface niches, and maintain effective colonization as root systems develop over time. Its contribution to successful rhizosphere colonization should be evaluated together with passive dispersal processes and bacterial interactions that shape microbial establishment under field conditions. Our proposed multi-layer engineering framework emphasizes that motility traits should be optimized against specific soil, crop, and management contexts. Incorporating motility related parameters into existing strain screening and development pipelines has the potential to improve the consistency and predictability of biofertilizer performance. However, translating this framework into practice remains challenging. Standardized platforms capable of evaluating motility under field-relevant conditions are still lacking, and quantitative relationships between motility phenotypes and colonization success have yet to be validated (Outstanding Questions). Addressing these gaps will require integrating data from microfluidic, transparent-soil, and field-tracking platforms and applying machine-learning approaches to relate motility phenotypes to soil, crop, and management variables, thereby improving prediction of field colonization and performance from laboratory-derived phenotypes. Understanding and engineering bacterial motility offers a concrete and actionable entry point for narrowing the gap between laboratory-observed potential and field-level outcomes.

### Outstanding questions

- What is the quantitative relationship between bacterial motility speed and colonization success? Is there a minimum threshold of swimming or swarming velocity required for effective rhizosphere colonization across different soil types and moisture regimes?
- Can the capacity for motility mode switching be translated into measurable colonization advantages under field conditions? Which combinations of motility modes are most critical across different soil textures and plant root systems?
- How can conventional laboratory-based motility assays be redesigned to better predict field colonization performance under field conditions? What key soil physical parameters must be incorporated?
- Should motility traits be strategically distributed among members of a synthetic consortium? Is it more effective to include specialized motile carrier strains or to ensure baseline motility across all consortium members?
- Does the introduction of highly motile inoculants affect the structure and function of native soil microbial communities and ecosystem functioning?
- What trade-offs exist between motility and other beneficial traits such as stress tolerance, biofilm formation, and metabolite production? To what extent can these trade-offs be decoupled through strain selection or engineering?

## Data Availability

No new data were generated or analysed in this article. All data discussed are available in the referenced publications.
